# Response surface-based media optimization for astaxanthin production in *Corynebacterium glutamicum*

**DOI:** 10.3389/fbioe.2025.1516522

**Published:** 2025-03-11

**Authors:** Florian Meyer, Ina Schmitt, Volker F. Wendisch, Nadja A. Henke

**Affiliations:** Genetics of Prokaryotes, Faculty of Biology and Center for Biotechnology (CeBiTec), Bielefeld University, Bielefeld, Germany

**Keywords:** astaxanthin, carotenoids, media optimization, design of experiment, *Corynebacterium glutamicum*

## Abstract

**Introduction:**

Astaxanthin is a C40 carotenoid that is used in animal feeds or cosmetics. Due to its high antioxidant property it is used for, e.g., anti-aging formulations and due to its intense red color it is used, e.g., in animal feed. While about 95% of commercial astaxanthin is currently chemically synthesized from fossil sources, the interest in natural and sustainable astaxanthin is growing. *Corynebacterium glutamicum*, an attractive host used in large-scale processes, e.g., industrial amino acid production, has been engineered for astaxanthin production.

**Methods:**

Here, a design of experiment (DoE) approach was applied to optimize the standard minimal medium for astaxanthin production. The concentrations of carbon, nitrogen and phosphorus sources, magnesium, calcium, the iron chelator protocatechuic acid, the vitamin biotin, and the trace metals were varied and astaxanthin production was evaluated.

**Results and discussion:**

By increasing the concentration of iron and decreasing that of manganese especially, it was possible to increase astaxanthin titers from 7.9 mg L^−1^–39.6 mg L^−1^ in a micro cultivation system and from 62 mg L^−1^–176 mg L^−1^ in a fed-batch fermentation.

## 1 Introduction

Astaxanthin is a C40 carotenoid with many applications in animal feed, healthcare and cosmetics. The latter applications mainly benefit from its high antioxidant (Edge et al., 1997; Krubasik et al., 2001; Henke et al., 2017b) and anti-inflammatory properties (MacDermid et al., 2012). Astaxanthin is responsible for the coloration of animal-derived food products like salmon flesh, lobsters and shrimp. These animals are incapable of synthesizing astaxanthin themselves, but instead acquire it from their feed sources, i.e., phytoplankton capable of synthesizing this carotenoid. In aquaculture, astaxanthin is added as a feed supplement to gain intense flesh coloring (Lorenz and Cysewski, 2000).

Industrial astaxanthin production requires petrochemical resources. Approximately 95% of commercially available astaxanthin is produced by (petro)chemical synthesis (Lorenz and Cysewski, 2000; Zhang et al., 2020). The remainder is obtained *via* extraction from microorganisms, mostly from *Phaffia rhodozyma* or *Haematococcus pluvialis* (Lorenz and Cysewski, 2000; Molino et al., 2018).

Recently, research to establish new producer organisms has gained momentum (Zhou et al., 2015; Henke et al., 2016; Lu et al., 2017). Among these, *Corynebacterium glutamicum* is especially promising. It has been used safely for more than 50 years in industrial biotechnology, for the production of amino acids like l-glutamate and l-lysine at the million ton scale (Zahoor et al., 2012). As an established workhorse in the food and feed industries it grows to high cell densities (Knoll et al., 2007) and possesses a high growth rate (Grünberger et al., 2013; Unthan et al., 2014). Additionally, its physiology is well understood, and an elaborated genetic tool box is at hand (Kalinowski et al., 2003; Wendisch et al., 2006; Baumgart et al., 2013; Cankar et al., 2023). With regards to a potential role as an astaxanthin producer, *C. glutamicum* is naturally pigmented due to biosynthesis of the C50 carotenoid decaprenoxanthin (Krubasik et al., 2001). Since both astaxanthin and decaprenoxanthin derive from the common precursor lycopene, systems metabolic engineering approaches were followed to engineer this bacterium for the production of other carotenoids (Krubasik et al., 2001; Heider et al., 2014a; Heider and Wendisch, 2015; Henke et al., 2016). To enable astaxanthin production, conversion of lycopene to decaprenoxanthin was abolished by deletion of the *crtY*_*e*_ *Y*_*f*_ *Eb* gene cluster, coding for lycopene elongase and flavuxanthin cyclase (Heider et al., 2014a). Subsequent heterologous expression of the genes *crtY*, *crtZ* and *crtW* coding for lycopene β-cyclase, β-carotene hydroxylase and β-carotene ketolase, respectively, enabled conversion of lycopene to astaxanthin (Heider et al., 2014b; Henke et al., 2016). The two terminal enzymes CrtZ and CrtW were then combined to the fusion protein CrtZ∼W obtaining the strain ASTA* (Henke et al., 2016; Henke and Wendisch, 2019).

To further optimize the process conditions in which these generated strains are cultivated, classically one factor at a time optimizations were used. As indicated by the name, this method changes one factor, i.e., one component of a medium, at a time, optimizes it and then goes on to the next component. However, this approach is time and ressource intensive and is also incapable of identifying combinatorial effects between multiple factors (Czitrom, 1999). This method has, for example, been successfully used in optimizing astaxanthin production in *P. rhodozyma* and increased titers by 92% (Ramírez et al., 2001).

Recently, process parameters of our astaxanthin fed-batch process were also improved using the design of experiment (DoE) methodology resulting in astaxanthin titers of 64 mg L^−1^ (Meyer et al., 2023). This was primarily achieved by shifting the pH of the process from 7 to 8 and decreasing the aeration rate. While the biological background of these effects is yet unclear, we speculated that it is linked to dissolved CO_2_ concentrations and iron availability. Transfer of the optimized process parameters to a better astaxanthin producing strain called ASTA** led to the production of 103 mg L^−1^ of astaxanthin (Göttl et al., 2024).

Up to date, comprehensive media optimization has not yet been performed. Previously, its potential became apparent with the observation that the addition of 2% (w/v) of potassium acetate (K-acetate) increased the astaxanthin production significantly (Henke and Wendisch, 2019). Many *C. glutamicum* studies use CGXII medium that was originally formulated for the production of lysine, an amino acid with two amino groups, thus, it contains high concentrations of nitrogen sources (Keilhauer et al., 1993). However, it was tempting to speculate that astaxanthin production could be improved with a tailored media composition as this compound contains no nitrogen and its biosynthesis differs from lysine biosynthesis. Thus, media composition experiments were conducted using a DoE approach to identify important media components and to increase product titers. As a result, we were able to formulate a mineral salt medium optimized for the production of xanthophylls. Specifically, astaxanthin titers increased five-fold in batch cultures and 2.5-fold in fed-batch fermentations, while requiring only relatively small compositional changes.

## 2 Materials and methods

### 2.1 Organism and cultivation conditions

The carotenoid producing strains of *C. glutamicum* and their respective plasmids used in this study are shown in Table 1.

**TABLE 1:** Overview over plasmids and *C. glutamicum* strains used in this study.

Strain	Description	References
ASTA*	BETA4 pSH1::*crtZ*_*FP*_∼*W*_*FP*_	Henke and Wendisch (2019)
BETA4	β-carotene producing derivative of *C. glutamicum*	Henke et al. (2016)
BETA6	BETA4 Δ*idi*::P_syn_-*idsA*-*idi*	Göttl et al. (2024)
BETA4 (pECXT::P_syn_-*crtZ*_*FP*_)	Hydroxylates β-carotene to β-cryptoxanthin and zeaxanthine	This work
BETA4 (pECXT::P_syn_-*crtW*_*FP*_)	Ketolates β-carotene to echinenone and canthaxanthin	This work
MB001 Δ*crtR*	Deletion of regulator *crtR* (cg0725) Increased production of decaprenoxanthine	Henke et al. (2017a)

Plasmid		
pSH1::*crtZ*_*FP*_ *∼W*_*FP*_	Fusion protein of β-carotene hydroxylase *crtZ* and ketolase *crtW* of *Fulvimarina pelagii*	Henke and Wendisch (2019)
pECXT::P_syn_-*crtZ*_*FP*_	β-carotene hydroxylase *crtZ* of *Fulvimarina pelagii*	Göttl et al. (2024)
pECXT::P_syn_-*crtW*_*FP*_	β-carotene ketolase *crtW* of *Fulvimarina pelagii*	Göttl et al. (2024)

All pre-cultures were first performed on LB (Sambrook et al., 1989) agar plates with appropriate antibiotics. These were incubated for 48 h at 30°C and in darkness, after which a single colony was used to inoculate 10 mL LB liquid medium with 10 g L^−1^ of glucose added. This first pre-culture was incubated on a rotary incubator at 120 rpm for 20 h at 30°C in the dark. Afterwards 1% (v v^−1^) of the pre-culture, with optical densities between 15 and 20, was transferred to 50 mL of CGXII medium (Keilhauer et al., 1993; Unthan et al., 2014) for flask and BioLector experiments, or 200 mL for fermentations. 40 g L^−1^ of glucose was added in each case as the carbon source unless stated otherwise (Keilhauer et al., 1993). Afterwards, the cultivation continued for another 24 h under the same conditions as before. These cultures were then used as pre-cultures for cultivations in FlowerPlates in the BioLector cultivation system (m2p-labs GmbH, Baesweiler, Germany), or for shaking flasks or bench-top stirred bioreactor experiments. For all flask and fermenter cultures, optical density measurements were performed using a UV-1202 spectrophotometer (Shimadzu, Germany, Duisburg). Recombinant *C. glutamicum* strains were constructed by transformation of the strain BETA4 with the plasmids pECXT::P_syn_-*crtZ*_*FP*_ and pECXT::P_syn_-*crtW*_*FP*_ accordingy by electroporation as described previously (van der Rest et al., 1999). Antibiotics, i.e. 25 μg mL^−1^ kanamycin and/or 5 μg mL^−1^ tetracycline were used in both solid and liquid media for strains carrying plasmids.

### 2.2 Response-surface methodology

Response-surface methodology (RSM) experiments were performed in the BioLector system. The FlowerPlates used contain 48 wells, which were filled with 980 µL of medium each. For inoculation, the pre-culture was centrifuged for 10 min at 4,000 × g and resuspended in the CGXII base, consisting only of urea, ammonium sulfate, di-potassium hydrogen phosphate and potassium di-hydrogen phosphate so that the final optical density at 600 nm was 50. After this, 20 µL of this suspension were then used to inoculate each well of the FlowerPlate.

Multiple response surface experiments were performed. The first experiment aimed at the determination of optimal concentrations for the macronutrients of the CGXII medium, i.e., urea, ammonium sulfate and both potassium phosphate salts as well as the carbon sources glucose and potassium acetate. Glucose was always added in the form of glucose monohydrate, however all concentrations are given with regard to the water-free glucose fraction. The second experiment was based on preliminary results from this first set of experiments and aimed to ascertain the effects of the micronutrients, i.e., MgSO_4_, CaCl_2_, biotin, protocatechuic acid (3,4-dihydroxybenzoic acid, PCA) and the trace metal solution. In the third experiment, the effects of the five trace metals contained in the solution, i.e., FeSO_4_, MnSO_4_, ZnSO_4_, CuSO_4_ and NiCl_2,_ were tested separately.

In general, all macronutrients were prepared as separate stock solutions at 10 x concentration (Table 2). These were combined for the different media compositions to their desired concentrations and filled up to 1 mL with sterile water. As CGXII contains both 1 g L^−1^ KH_2_PO_4_ and 1 g L^−1^ K_2_HPO_4_, we converted their total concentration to 13.05 mM phosphate. We then used this value to combine both phosphates into a pH 7 stock solution containing 130.5 mM phosphate (Table 2). Trace elements were used at their standard CGXII concentrations (Keilhauer et al., 1993; Unthan et al., 2014) for the first experiment.

**TABLE 2:** Overview of the stock solutions of the separately prepared media components for the design of experiment approaches. Glucose was added as glucose monohydrate, however all concentrations given are only for the water-free glucose.

Compound	Concentration of stock solution	Notes	Final concentration range for DoE	Sterilized by
Macronutrients				
Urea	50 g L^−1^		0–5 g L^−1^	Filtration
Ammonium sulfate	200 g L^−1^		10–30 g L^−1^	Autoclave
K_2_HPO_4_ KH_2_PO_4_	2.273 g L^−1^ 1.776 g L^−1^ Combined: 130.5 mM PO_4_^3-^	Both solutions combined to a pH of 7	2–5 g L^−1^	Autoclave
MOPS	420 g L^−1^	Adjusted to pH 7 with KOH	42 g L^−1^	Filtration
Glucose	400 g L^−1^		20–60 g L^−1^	Autoclave
K-Acetate	200 g L^−1^		0–20 g L^−1^	Filtration
Micronutrients				
MgSO_4_ × 7 H_2_O	250 g L^−1^		0–500 mg L^−1^	Autoclave
CaCl_2_	10 g L^−1^		0–20 mg L^−1^	Autoclave
Biotin	200 mg L^−1^		0–0.4 mg L^−1^	Filtration
Protocatechuic acid	30 g L^−1^		0–60 mg L^−1^	Filtration
1,000 x Trace metal solution	Combination of trace metals in concentrations given below		0 x – 2 x	Autoclave
Trace metals				
First experiment				
FeSO_4_ × 7 H_2_O	10 g L^−1^		0–20 mg L^−1^	Filtration
MnSO_4_ × H_2_O	10 g L^−1^		0–20 mg L^−1^	Filtration
ZnSO_4_ × 7 H_2_O	1 g L^−1^		0–2 mg L^−1^	Filtration
CuSO_4_	200 mg L^−1^		0–0.4 mg L^−1^	Filtration
NiCl_2_ × 6 H_2_O	20 mg L^−1^		0–0.04 mg L^−1^	Filtration
Second experiment				
FeSO_4_ × 7 H_2_O	10 g L^−1^		11.68–28.3 mg L^−1^	Filtration
MnSO_4_ × H_2_O	10 g L^−1^		0.84–9.16 mg L^−1^	Filtration
ZnSO_4_ × 7 H_2_O	1 g L^−1^		1.18 mg L^−1^	Filtration
CuSO_4_	200 mg L^−1^		0.017–0.18 mg L^−1^	Filtration
NiCl_2_ × 6 H_2_O	20 mg L^−1^		0.0146 mg L^−1^	Filtration

The experimental design and analysis was done using R 4.3.0 (R Core Team, 2023) and the rsm package version 2.10.3 (Lenth, 2009). The design of the first experiment was a fractional factorial central composite design and consisted of 2^4^ different media compositions by aliasing the effects of phosphate with the combined effects of the other four media components. All conditions were tested in technical triplicates. The concentration ranges for all compounds are shown in Table 2.

These 48 conditions were tested on a single FlowerPlate, while triplicate center and face-centered star points were done on a second plate. To allow the determination of block effects three factorial points from the first plate were repeated in triplicate on the second plate. Face-centered star points were chosen since acetate and urea were already at a concentration of 0 g L^−1^ and thus no further decrease was possible to establish star points at a further distance. Additionally, concentrations of glucose higher than 60 g L^−1^ would have likely led to toxic effects. At the star points all other concentrations were kept the same as they were at the center points. The plates were then cultivated for 100 h at 30°C and 1,100 rpm. Optical densities were measured automatically every 15 min, as well as once photometrically at the end as described before. At that time, also 0.5 mL of each well were subjected to carotenoid extraction and subsequent HPLC analysis.

The results of the RSM were then analyzed and a set of new cultivations based on the steepest ascent of astaxanthin concentration was performed in the FlowerPlates as already described.

The subsequent second and third experiment were then performed based on the preliminarily results of this first RSM and resulted in the medium composition CGAST1 consisting of 12.1 g L^−1^ K-acetate, 42.6 g L^−1^ glucose, 19.3 g L^−1^ ammonium sulfate, 3.5 g L^−1^ urea and 3.8 g L^−1^ phosphate.

For the second experiment, concentrations of the micronutrients were varied between 50% and 150% of standard CGXII concentrations for the central design and 0% and 200% for the star points. Again, 2^4^ different combinations were tested in a fractional factorial central composite design by aliasing the effect of the trace metal solution with the combined effect of the remaining four media components. Cultivations of the cube and star points were performed in technical duplicates. For the center points, eight replicates were performed for the first cultivation, containing the cube portion of the design and four replicates for the cultivation containing the star portion of the design. Again, final optical densities were measured and 0.5 mL of each well were harvested for carotenoid analysis.

Finally, in a third experiment separate solutions of the trace metals, FeSO_4_, MnSO_4_, ZnSO_4_, CuSO_4_ and NiCl_2_ were prepared and their effects were tested by setting concentrations to 0% or 200% of their initial concentrations for the star points, 50% and 150% for the cube portion of the model and the standard CGXII concentrations for the center points. Center points had all concentrations at their standard CGXII values, i.e. 100%, while the star points had one concentration at 0% or 200% while all others were kept at 100%. These experiments were performed in a single FlowerPlate and contained 13 center points, star points without replicates and the cube portion consisting of 2^4^ runs. The effect of nickel was confounded with the combined effect of the other four trace metals, thus again leading to a fractional factorial central composite design. Finally, a second central composite design was utilized to further optimize the trace metal concentrations based on the first experiment. In this both NiCl_2_ × 6 H_2_O and ZnSO_4_ × 7 H_2_O concentrations were set to 0.0144 mg L^−1^ and 1.18 mg L^−1^, respectively while the concentration of FeSO_4_ × 7 H_2_O was varied between 28.3 mg L^−1^ and 11.7 mg L^−1^ for the star points and 15, 20 and 25 mg L^−1^ for the cube portion and center points respectively. MnSO_4_ × H_2_O was varied between 0.8 and 9.2 mg L^−1^ for the star points, 5 mg L^−1^ for the center points and 2.5 and 7.5 mg L^−1^ for the cube portion of the design. CuSO_4_ was varied between 0.017 mg L^−1^ and 0.183 mg L^−1^ for the star portion, 0.1 mg L^−1^ for the center points and 0.1 and 0.15 mg L^−1^ for the cube portion of the design. All utilized stock solutions as well as their final concentration ranges are shown in Table 2. All designed experiments with all tested conditions are shown in Supplementary Table S3.

### 2.3 Extraction of carotenoids and quantification by HPLC

The carotenoid contents were determined by harvesting of 0.5 mL of culture broth at the indicated time points (given in the results section) after centrifugation for 10 min at 4,000 Χ g. The supernatant was discarded and the cell pellet resuspended in 1 mL of methanol:acetone (7:3) and incubated for 30 min at 60°C, shaking at 600 rpm. Afterwards, the extract was separated from cell debris by centrifugation at 21.000 Χ g for 10 min and the supernatant collected for HPLC measurement. The extraction was repeated until the cell pellet was completely white, to achieve a complete removal of all carotenoids (Henke and Wendisch, 2019). The HPLC analysis was performed using an Agilent 1,200 series system (Agilent Technologies, Waldbronn, Germany), equipped with a C18 reverse phase column (LiChrospher 100 RP18 EC-5, 125 × 4 mm) and precolumn (LiChrospher 100 RP18 EC-5, 40 × 4 mm). Both column and pre-column were obtained from Merck KGaA (Darmstadt, Germany). Detection of the carotenoids was achieved using a diode array detector measuring and recording at wavelength 471 nm. Additionally, the detector recorded the whole spectrum between 250 nm and 500 nm. Authentic standards of 3-hydroxyechinenone, adonirubin, adonixanthin, canthaxanthin (all from CaroteNature GmbH, Münsingen, Switzerland), β-carotene (Fisher Scientific, Schwerte, Germany), β-cryptoxanthin, lycopene, zeaxanthin (all from Carl Roth GmbH + Co. KG, Karlsruhe, Germany), astaxanthin (Cayman Chemical, Ann Arbor, USA) and echinenone (Merck KGaA, Darmstadt, Germany) were measured to identify retention times and absorption coefficients for quantification.

Carotenoids were separated using a gradient between 9:1 methanol:water (A) and pure methanol (B) at 1.5 mL min^−1^ in the form of the following linear gradient: 0 min B: 0%, 10 min B: 100%, 32.5 min B: 100%, 33 min B: 0% (Henke and Wendisch, 2019).

### 2.4 Fed-batch fermentation

Fed-batch fermentations were performed as described previously (Meyer et al., 2023). Briefly, fermentations were performed in 3.7 L KLF glass bioreactors (Bioengineering AG, Wald, Switzerland) with a maximum of 2 L of working volume. The stirrer-to-reactor diameter ratio was 0.39 and the aspect ratio was 2.6:1.0. Three six-bladed Rushton turbines were installed on the axis with distances of 6, 12 and 17 cm from the bottom of the reactor. Fermentations were performed at a continuous temperature of 30°C and pH was kept constant at 8 ± 0.1 using 10% phosphoric acid and 25% ammonia. The aeration rate was set to 0.25 vvm initially and increased manually when required to keep oxygen saturation above 0%. The starting volume was 1 L of *C. glutamicum* HCDC medium (Knoll et al., 2007) containing: 10 g L^−1^ glucose as carbon source, 19.75 g L^−1^ KH_2_PO_4_, 39.5 g L^−1^, 10 g L^−1^ (NH_4_)_2_SO_4_, 6.67 g L^−1^ MgSO_4_ × 7 H_2_O, 0.27 g L^−1^ CaCl_2_ and a trace metal composition given in Table 3. The feed consisted of 600 g L^−1^ glucose and was constantly stirred at room temperature to prevent phase separation. The stirrer rate was automatically increased stepwise, whenever the dissolved oxygen concentration (rDOS) fell below 30%. Feed was added whenever the rDOS rose above 60%, indicating that the cells had consumed all available carbon source, and was stopped when rDOS fell again below 60%. Samples were taken automatically every 3 h and cooled to 4°C until further use for optical density determination and carotenoid quantification. A mechanical foam breaker was installed on the stirring axis, 22 cm above the fermenter bottom and up to 30 mL of AF204 antifoam agent were automatically added based on a foam probe signal to control foaming.

**TABLE 3:** Overview over previously published and newly developed media compositions used in this study. The table shows the compositions of the standard CGXII medium (Keilhauer et al., 1993; Unthan et al., 2014), the fed-batch HCDC medium (Knoll et al., 2007) and the media optimized for astaxanthin production CGAST1, CGAST2 and the corresponding optimized trace metal composition. The micronutrient composition used was either that of standard CGXII for batch cultivations in the milliliter scale, or the standard Knoll HCDC composition when cultivating on the 2 L scale in a fed-batch process. The trace metal composition was either the standard CGXII or optimized batch composition on the milliliter scale, or the standard HCDC or optimized fed-batch composition for the fed-batch fermentation scale.

Macronutrients	CGXII [g L^−1^] (Keilhauer et al., 1993; Unthan et al., 2014)	CGAST1 [g L^−1^]	CGAST2 [g L^−1^]	Knoll HCDC medium [g L^−1^] (Knoll et al., 2007)
Glucose	40	42.6	50.5	10
K-Acetate	0	12.1	12.7	0
Urea	5	3.5	7.4	0
Ammonium sulfate	20	19.3	9.6	10
K_2_HPO_4_	1	1.9	0.95	39.5
KH_2_PO_4_	1	1.9	0.95	19.75

Micronutrients	CGXII composition used for batch cultivations [mg L^−1^]	Knoll HCDC composition used for fermentations [mg L^−1^]
MgSO_4_ × 7 H_2_O	250	6,670
CaCl_2_	10	270
Biotin	0.2	5.3
Protocatechuic acid	30	800

Metal salt	CGXII [mg L^−1^]	Optimized batch composition	Knoll HCDC medium [mg L^−1^]	Optimized fermentation medium [mg L^−1^]
FeSO_4_ × 7 H_2_O	10	20	530	400
MnSO_4_ × 2 H_2_O	10	5	270	100
ZnSO_4_ × 7 H_2_O	1	1.18	27	23.6
CuSO_4_	0.2	0.1	5.3	2
NiCl_2_ × 6 H_2_O	0.02	0.0146	0.53	0.292

## 3 Results

### 3.1 Response surface methodology based media optimization

Media optimization was performed using a response surface methodology and included macro- and micronutrient optimization, with a special focus on the trace metals.

#### 3.1.1 Macronutrients optimization

In order to identify important media components and to increase astaxanthin titers of *C. glutamicum* ASTA*, a design of experiment (DoE) approach was followed. The macronutrients of CGXII, ammonium sulfate, urea, phosphate and additionally the carbon sources glucose and K-acetate, were varied in their concentrations in the DoE. Except for phosphate, all of these components showed a positive correlation with biomass formation, although a negative two-factor interaction was identified between K-acetate and urea. Phosphate showed no influence on the optical density in the range analyzed (Supplementary Table S1; Supplementary Figure S1).

**FIGURE 1:**
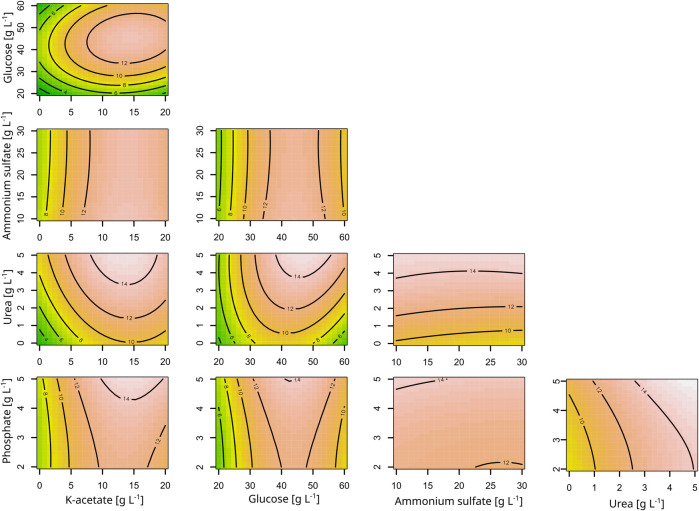
Contour plots of the response surface model for the effects of the macronutrients of CGXII on astaxanthin titers. Astaxanthin titers are given as mg L^-1^ and shown as labels in the plots. All other concentrations are given as g L^-1^. The response surface is sliced at Block = 1.47, glucose = 40.67, K-acetate = 9.67, ammonium sulfate = 19.00, urea = 2.58, phosphate = 3.55.

**FIGURE 2:**
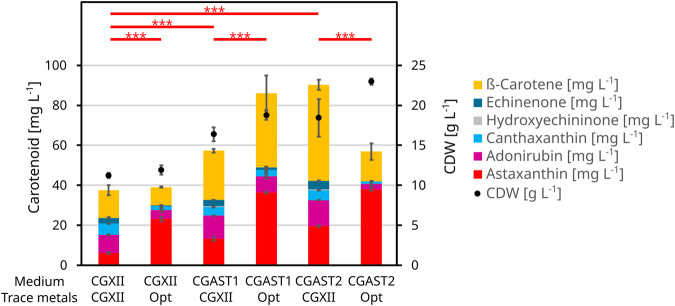
BioLector experiment to show the effects of the optimization of the CGXII base medium and its trace metals on astaxanthin formation and CDW. All cells were grown in 1 mL of medium at 1,100 rpm and 30°C for 48 h. The strain used was *C. glutamicum* ASTA*. The medium was either CGXII or one of two optimized compositions called CGAST1 and CGAST2. These base media consist of glucose, acetate, ammonium sulfate, urea and phosphate in differing concentrations. The trace metals are either at their standard CGXII levels or the optimized composition derived in this study. The trace metals comprise of FeSO_4_, MnSO_4_, ZnSO_4_, CuSO_4_ and NiCl_2_. Other media components like biotin and PCA were kept at their initial CGXII concentrations.

Regarding the astaxanthin titers, all macronutrients except for ammonium sulfate had a positive influence. For both astaxanthin and CDW, the second-order model had a significant lack-of-fit. However, all model terms were highly significant for the astaxanthin model and all but the quadratic terms were significant for the CDW model (Supplementary Table S1). The corresponding contour plots are shown in Figure 1. Since the astaxanthin titer was the important response parameter, further experiments were done with the medium optimized for astaxanthin production. A steepest ascent and a stationary point analysis were performed as the stationary point identified was only a saddle point. The steepest ascent (data not shown) led to a medium composition of: 12.1 g L^−1^ K-acetate, 42.6 g L^−1^ glucose, 19.3 g L^−1^ ammonium sulfate, 3.5 g L^−1^ urea and 3.8 g L^−1^ phosphate. This medium was named CGAST1 and used for the subsequent optimizations of micronutrients and trace metals. Only after these further experiments were started, it was decided to test the medium composition at the saddle point as well. The saddle point was identified at 12.7 g L^−1^ K-acetate, 50.5 g L^−1^ glucose, 9.6 g L^−1^ ammonium sulfate, 7.4 g L^−1^ urea and 1.9 g L^−1^ phosphate. The resulting medium was named CGAST2. With both media it was possible to significantly increase astaxanthin titers 2.1 and 3.2-fold, respectively, from 5.6 ± 0.4 mg L^−1^ to 12.1 ± 0.4 mg L^−1^ and 18.1 ± 4.2 mg L^−1^ (*p* values 1.6*10^−5^ and 6*10^−6^, respectively) (Figure 2). Furthermore, a clear increase in the total concentration of carotenoids produced was visible as they increased from 36.8 ± 3.2 mg L^−1^ in CGXII medium to 56.8 ± 8.1 mg L^−1^ and 89.3 ± 8.9 mg L^−1^ in CGAST1 and CGAST2 medium, respectively. The developed media compositions are shown in Table 3.

#### 3.1.2 Micronutrients optimization

**FIGURE 3:**
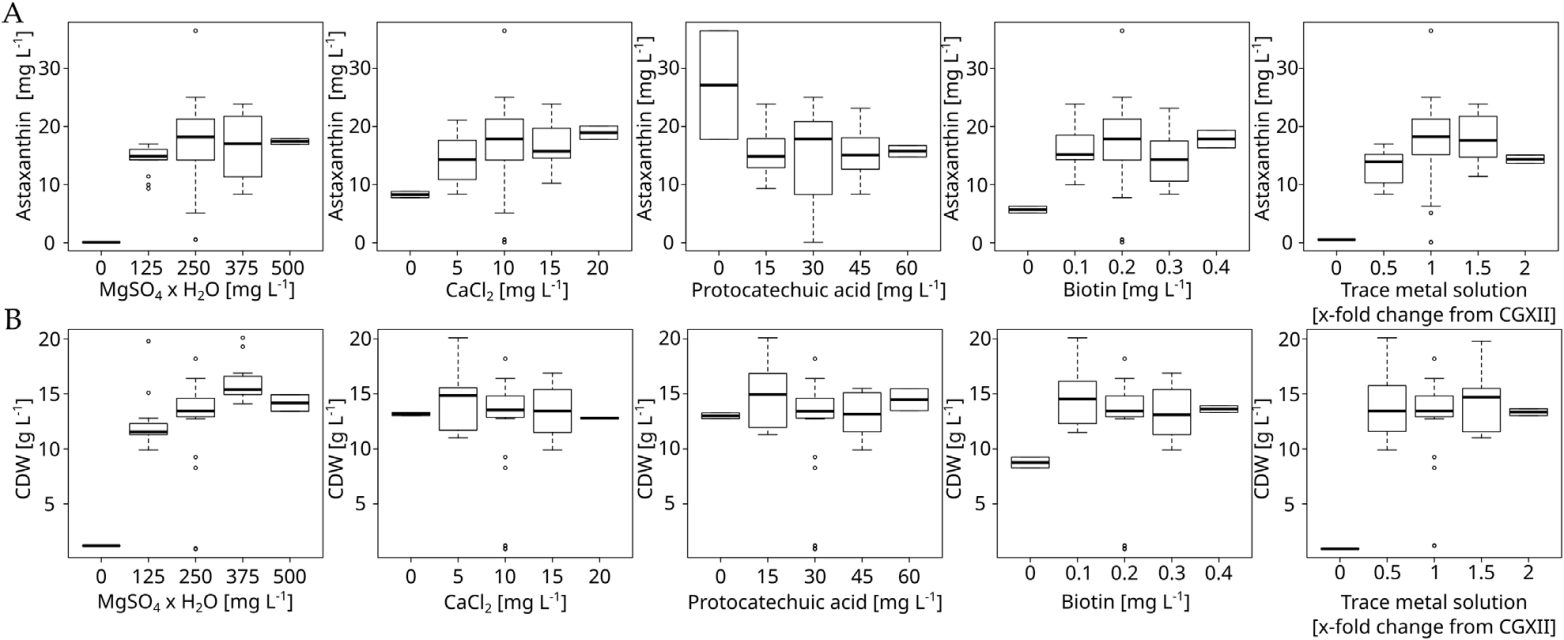
Boxplot of the micronutrient optimization experiment with *C. glutamicum* ASTA* in modified CGXII medium at 30°C, 1,100 rpm in a BioLector FlowerPlate. Concentrations are given as mg L^−1^ or x-fold change from default CGXII concentrations in the case of the trace metal solution. Shown are both astaxanthin **(A)** and biomass **(B)** titers.

After the macronutrients optimization, a second DoE experiment focusing on the remaining media components MgSO_4_, CaCl_2,_ biotin, PCA and the CGXII trace metal solution was performed. Two rounds of cultivations were performed with concentrations being varied between 0, 0.5, 1.0, 1.5 and 2.0 times their standard CGXII concentrations. 0 and 2.0 were used as star-points while 0.5, 1.0 and 1.5 were used for the cube portion of the experiment. The resulting boxplots are shown in Figure 3. These experiments showed that magnesium is clearly limiting the maximum attainable CDW. Furthermore, these results showed that removal of magnesium or trace metals leads to a complete growth arrest, while removing biotin leads to an observable, but smaller reduction in maximum CDW. These results were mirrored for astaxanthin titers, with a stronger reduction in production being observed under biotin removal as well as calcium removal. The resulting RSM indicated an optimum at 360 mg L^−1^ MgSO_4_ × 7 H_2_O (1.44 fold), 12.7 mg L^−1^ CaCl_2_ (1.27 fold) and 1.49 fold the initial amount of the trace metal solution. However, upon verifying this prediction in a 10 mL flask culture in both unmodified CGXII and CGAST2, it was observed that astaxanthin titers actually decreased at this medium composition (Supplementary Figure S2). While the RSM showed a non-significant lack of fit, it is still possible that the resulting optimum was skewed due to most of the data points being similar to each other, while only the extreme low side showed very strong effects. However, there appeared to be a quadratic relationship between the trace metal concentration and the astaxanthin titer present in the gathered data and the trace metals showed the strongest influence overall on astaxanthin titers in the generated model.

#### 3.1.3 Trace metal optimization

As the optimization of the micronutrients in the medium did not improve astaxanthin titers, but showed a possible effect from the trace metal concentrations on product titers, the trace metals and their effects on astaxanthin production were investigated in more detail. The previously used trace metal solution contains five metal salts, namely, FeSO_4_ × 7 H_2_O, MnSO_4_ × H_2_O, ZnSO_4_ × 7 H_2_O, CuSO_4_ and NiCl_2_ × 6 H_2_O. Separately prepared solutions allowed to change their concentrations independently. Concentrations were varied between 0, 0.5, 1.0, 1.5 and 2.0 times their standard CGXII concentrations (Table 2) and CGAST1 was used as the base medium for this experiment.

**FIGURE 4:**
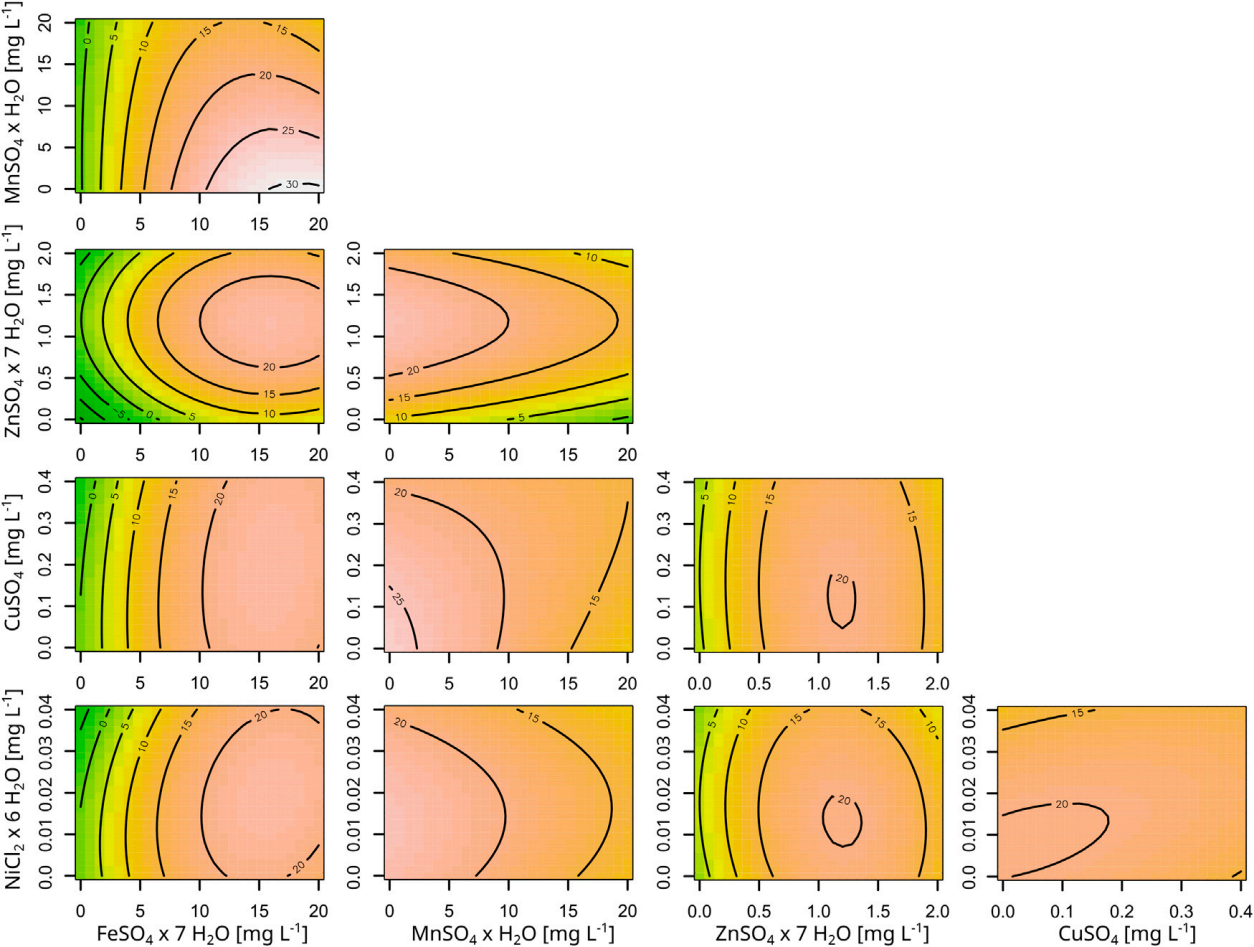
Contour plots of the RSM for the five trace metals and their effect on astaxanthin titers. All concentrations are given as mg L^-1^ and the astaxanthin titers are shown as labels in the plots. The model is based on a five factor RSM, performed in a BioLector cultivation system using strain *C. glutamicum* ASTA* at 30°C and 1,100 rpm.

The resulting RSM is shown in Supplementary Table S2 and the corresponding contour plots are shown in Figure 4. Furthermore, Supplementary Figure S3 shows the data as a boxplot. This experiment showed the different importance of the various metals for *C. glutamicum*, as growth without iron was completely inhibited and the final OD severely reduced to 50% without zinc. Contrary, no negative effects were observed on growth from removing copper, manganese or nickel. Only upon transfer of cells from manganese-free medium to the same medium again did the cells show a growth limitation of 73% or a CDW of 2.45 ± 1.1 g L^−1^. With regard to astaxanthin production, significant effects were observed for FeSO_4_, MnSO_4_ and ZnSO_4_ but not for CuSO_4_ and NiCl_2_. Furthermore, there is a significant negative interaction between iron and manganese, where high titers of manganese diminish the positive effect from high iron concentrations. The identified optima for ZnSO_4_ × 7 H_2_O and NiCl_2_ × 6 H_2_O were well within the experimental space and were thus set to 1.18 mg L^−1^ and 0.0146 mg L^−1^, respectively. However, both the optima of iron and manganese were at the edge of the experimental space and there appeared to be a potential effect when leaving out copper entirely. Thus, a second experiment was performed to further analyze astaxanthin production under a more limited set of trace metal concentrations while keeping the other conditions identical. Zinc and nickel were set to their optimal concentrations while FeSO_4_ × 7 H_2_O was tested between 11.68 mg L^−1^ and 28.32 mg L^−1^, MnSO_4_ × H_2_O between 0.84 mg L^-1^ and 9.16 mg L^−1^ and CuSO_4_ between 0.18 mg L^−1^ and 0.017 mg L^−1^ (Table 2). In this experiment a further improvement was found at the new center point at an FeSO_4_ × 7 H_2_O concentration of 20 mg L^−1^, MnSO_4_ × H_2_O concentration of 5 mg L^−1^ and CuSO_4_ concentration of 0.1 mg L^−1^ (Table 3), resulting in an astaxanthin titer of 35.9 ± 0.0 mg L^−1^. This trace metal composition was then combined with the previously established medium compositions CGAST1 and CGAST2 in another BioLector cultivation, which resulted in a titer of 33.6 ± 1.2 mg L^−1^ in CGAST1 and 35.0 ± 1.1 mg L^−1^ in CGAST2. While the titers were not significantly different, the total concentration of carotenoids was significantly reduced in CGAST2 to 54.2 ± 3.9 mg L^−1^ from 83.8 ± 2 mg L^−1^ in CGAST1 leading to a potentially higher purity of isolated astaxanthin, but also to a lower pool of carotenoids that can be converted to astaxanthin by future strain engineering (Figure 2).

### 3.2 Influence on production of intermediates of astaxanthin biosynthesis

**FIGURE 5:**
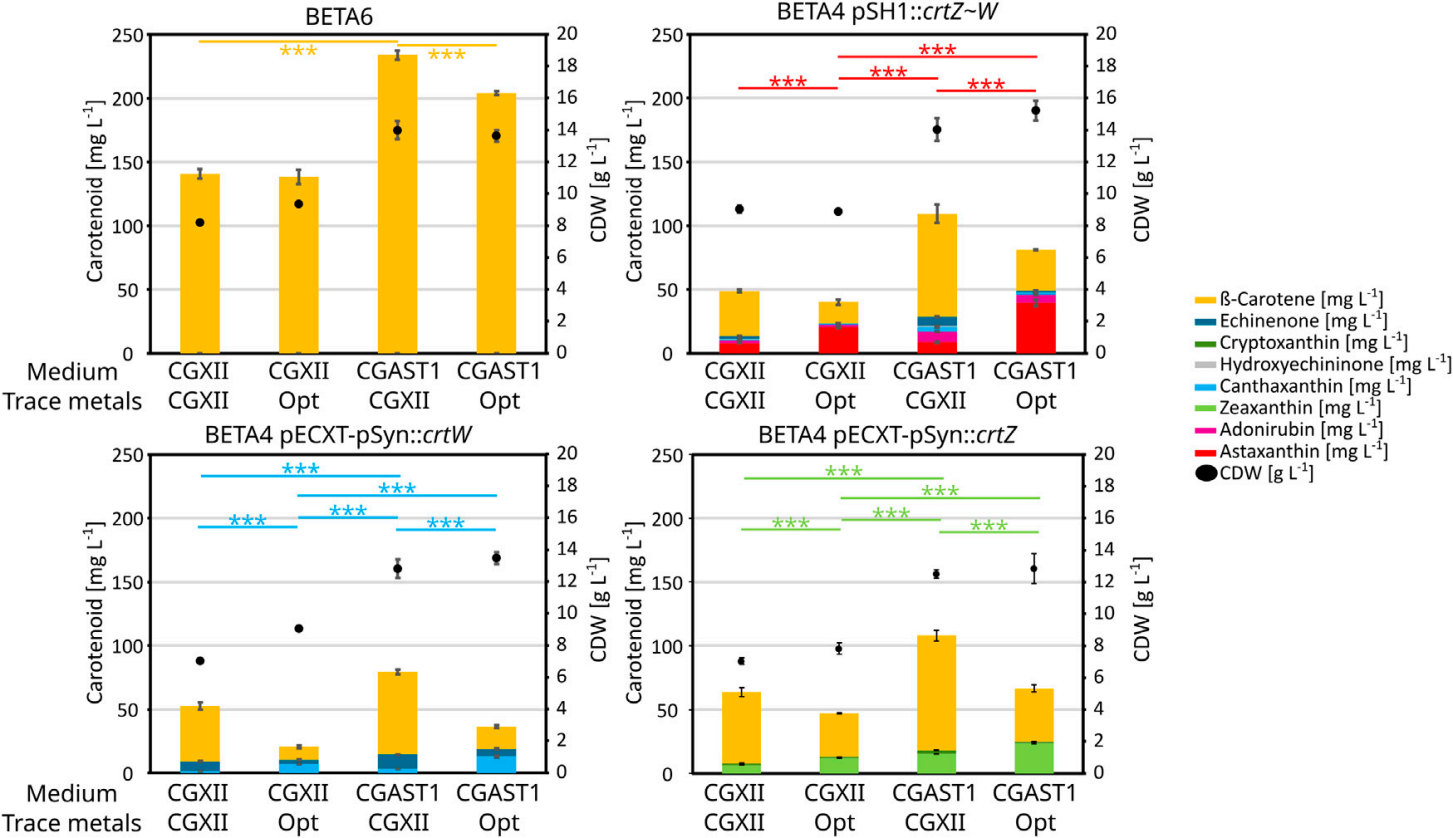
Carotenoid titers and CDW from an experiment performed in 1 mL of medium in BioLector FlowerPlates at 30°C and 1,100 rpm. Used *C. glutamicum* strains are given over each graph, carotenoids are shown in bar plots and CDW is shown as dots. All experiments were performed in triplicates and the standard deviation is shown. Medium compositions were either regular CGXII, CGXII with optimized trace metal composition, CGAST1 with the standard CGXII trace metal composition or CGAST1 with optimized trace metals. T-test significances are given for each main product in each graph.

To identify the source of the positive effects of these changes in the media composition, different carotenoid producing strains were analyzed (Figure 5): BETA6 (β-carotene), BETA4 pECXT-pSyn:*crtW* (echinenone and canthaxanthin), BETA4 pECXT-pSyn:*crtZ* (β-cryptoxanthin and zeaxanthin), as well as ASTA* (BETA4 pSH1:*crtZ∼W*) as the astaxanthin producing strain. The base medium optimization primarily increased total carotenoids and especially β-carotene, while the trace metals influenced conversion of β-carotene to the corresponding xanthophylls. Furthermore, changing the trace metal composition even shows a 12.5% reduction on β-carotene production by strain *C. glutamicum* BETA6 when combined with CGAST1 medium, or no effect in combination with CGXII. Contrary to this, canthaxanthin and zeaxanthin production were positively influenced by both the optimized trace metals and base medium and increased 8.2 and 3.6-fold, respectively. This effect stems primarily from the trace metal optimization in the case of β-carotene ketolation to canthaxanthin, as the change between CGXII to CGAST1 only increases titers 2.2-fold, while the change in trace metals changes it between 3.7 and 4.5-fold in CGXII and CGAST1, respectively. For β-carotene hydroxylation to zeaxanthin, both changes contributed about equally with trace metals increasing zeaxanthin titers by 1.8-fold and the change from CGXII to CGAST1 increasing it 2.3-fold. Furthermore, a reduction in total carotenoids, especially β-carotene, was observed when increasing titers of xanthophylls like astaxanthin, canthaxanthin or zeaxanthin were produced.

### 3.3 Influence on decaprenoxanthin production

**FIGURE 6:**
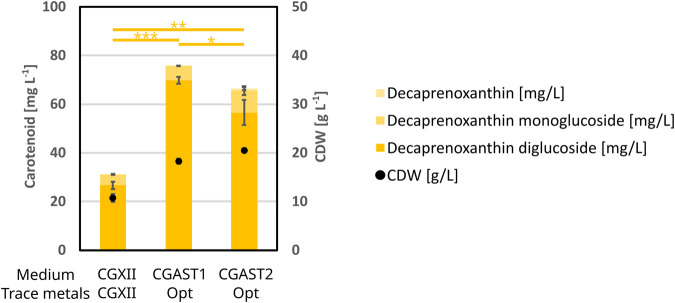
BioLector experiment to show the effects of the optimization of the CGXII base medium and its trace metals on decaprenoxanthin formation and CDW. All cells were grown in 1 mL of medium at 1,100 rpm and 30°C for 48 h. The strain used was *C*. *glutamicum* Δ*crtR*. The medium compositions were either regular CGXII or the optimized media CGAST1 or CGAST2 with the also optimized trace salt composition. All experiments were performed in triplicates and the standard deviation is shown. T-test significances for decaprenoxanthin are given as lines and stars.

While the media optimization had the explicit goal of improving astaxanthin titers, its influence on the production of the native C50 carotenoid decaprenoxanthin diglucoside was examined as well. To this end regular CGXII was compared against the optimized media CGAST1 and CGAST2 including the optimized trace salts. The results are shown in Figure 6. Overall, the optimized medium was also beneficial for decaprenoxanthin production, as its titer increased from 26.6 mg L^−1^ to 69.8 mg L^−1^ and 56.5 mg L^−1^ for CGAST1 and CGAST2, respectively. In part, this is due to the increase in CDW from 10.8 g L^−1^ to 18.3 g L^−1^ and 20.5 g L^−1^ in CGAST1 and CGAST2, respectively. However, in CGAST1 the mg g^−1^ CDW also increased significantly from 2.5 mg g^−1^ to 3.8 mg g^−1^, while no significant increase was observed in CGAST2.

### 3.4 Transfer of the optimized trace metal solution to high cell density cultivation medium

**FIGURE 7:**
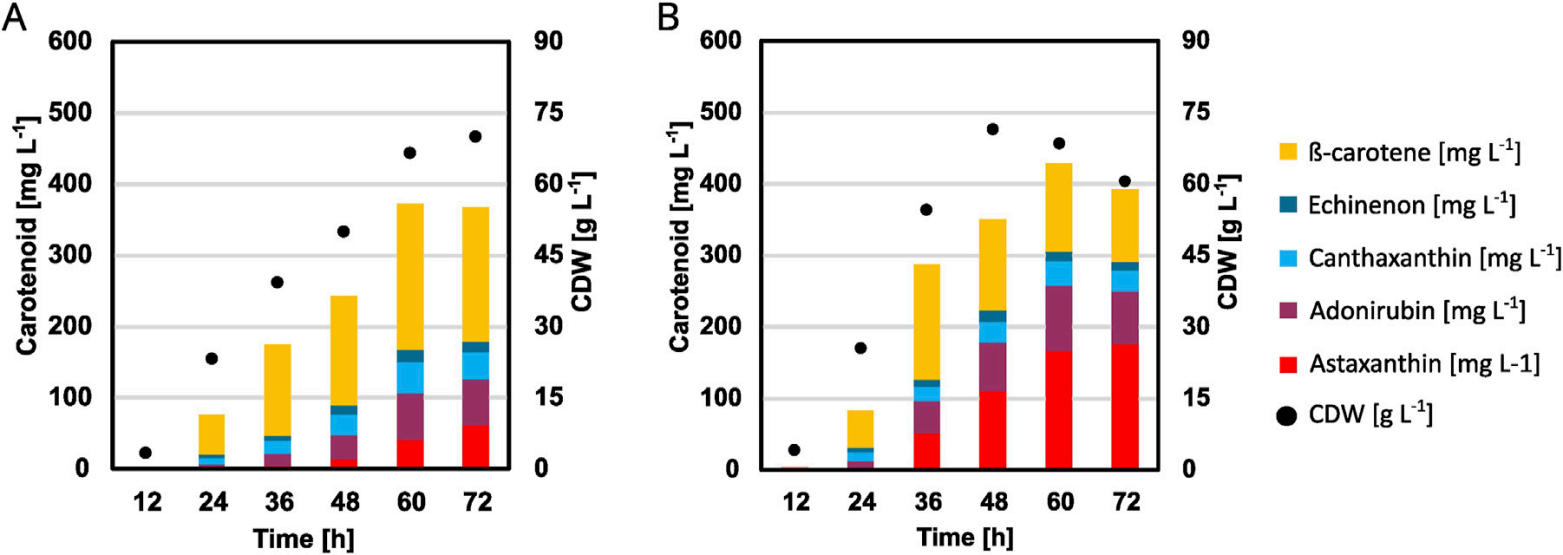
Fermentations of strain *C. glutamicum* ASTA* in a 2 L fed-batch reactor using 1 L of a modified Knoll HCDC medium with either the standard trace metal composition **(A)** or the composition optimized for cultivation in BioLector FlowerPlates **(B)**. A feed of 1 L 600 g L^−1^ glucose was added based on the pO_2_ signal. The process was performed at 30°C and pH 8 using 25% (v/v) ammonia and 10% (v/v) phosphoric acid.

To verify if the optimization of the microcultivation can also be transferred, an adapted high-cell density fed-batch medium was used with the optimized trace metal concentrations and applied in a 2 L fed-batch fermentation. As the fermentation base medium is optimized for high-cell density and contains no urea, the optimized base media are not really transferable to a larger scale, however the trace-metals can easily be transferred and also showed the higher effect on astaxanthin titers in the previous experiments. Furthermore, previously good results were obtained (Meyer et al., 2023) with the published HCDC medium by Knoll et al. (2007). Because of these reasons, it was decided to adapt the trace metal composition to the fermentation process. The previous experiments showed that especially the iron and manganese concentrations and their ratio to each other were important, thus these ratios were kept the same compared to the small-scale experiments while upscaling to the fermentation scale. However, the concentrations found in the milliliter scale are not enough to allow cell growth up to the typical densities of up to 100 g L^−1^ and thus needed to be increased. Additionally, the basal fermentation medium containing the trace metals is diluted 1:2 with feed solution, further increasing the need for higher metal ion concentrations. Therefore, 20 x the concentration that was used for the flask and BioLector experiments to account for both the doubling in volume (2x) due to the addition of feed and the higher expected cell densities (∼10x), while keeping the relative composition identical. This leads to overall lower metal concentrations compared to the previously used Knoll HCDC medium (Knoll et al., 2007). Most importantly, there is a large reduction in manganese and an increase in the ratio between iron and manganese (Table 3). Figure 7 and Supplementary Figure S4 show the resulting plots. Maximum CDW were quite comparable being 70 g L^−1^ for the control and 71.5 g L^−1^ for the optimized condition. Feed consumption was quicker using the optimized trace metals as well, as the feed solution was fully consumed after 51 h, compared to it not being fully consumed when the process was stopped after 72 h in the case of the control. This is also mirrored in the strain reaching its maximum CDW after 48 h, compared to 72 h for the control condition. Most importantly, the change in trace metals led to a 2.8-fold increase in astaxanthin from 62 mg L^−1^ to 176 mg L^−1^.

## 4 Discussion

In this work, a DoE approach was applied for the optimization of the minimal medium CGXII for the production of astaxanthin. This medium was originally designed for the production of large quantities of amino acids and therefore contains high concentrations of nitrogen in the form of urea and ammonium sulfate (Keilhauer et al., 1993). Since carotenoids are not nitrogenous, the expectation was that a significant improvement in the medium composition is possible. Similar approaches have been already performed for carotenoid production (Ramírez et al., 2001; Shaker et al., 2017). Furthermore, previously a similar approach was applied to optimize process parameters (Meyer et al., 2023) with good results.

The primary result in the current study is that (i) the mixture of carbon source, in this case glucose and K-acetate, influences carotenoid production in general and that (ii) the trace metals, specifically iron and manganese, affect the conversion from β-carotene to downstream carotenoids. Surprisingly, the concentration of phosphate and the two nitrogen sources had only a minor impact on astaxanthin production, while the choice of nitrogen source and concentration has been observed to be an important factor in other organisms, e.g., *P. rhodozyma* (Vustin et al., 2004; Ni et al., 2007)*.* For *C. glutamicum*, it might be possible to fully replace ammonium sulfate with urea to reduce media costs and to benefit from using urea that has shown a positive effect on astaxanthin production. This replacement has been tested previously in media for *C. glutamicum* but not specifically for carotenoid production (Yang et al., 2021). The presence of urea could have a benefit due to supplying CO_2_ during the initial stages of the cultivation, which will be discussed further down, or it could be due to differences in gene expression that have been observed by others. However, these latter analyses were performed between cultures grown in CGXII and R2 medium, which contains more changes than just the exchange of ammonium sulfate for urea (Yang et al., 2021).

The importance of acetate and glucose concentrations had been observed for astaxanthin production in *C. glutamicum* before, with the possible explanation that co-metabolization of acetate and glucose increases intracellular concentrations of important isoprenoid precursors like GAP and pyruvate, thus increasing carotenogenesis (Gerstmeir et al., 2003; Henke and Wendisch, 2019). The higher titers of precursor carotenoids could then lead to a higher titer of astaxanthin as well.

For the micronutrients, we observed that trace metals and magnesium are strictly necessary for growth, while calcium and PCA can be omitted, at least for a single round of cultivation. However, magnesium showed a much stronger correlation with the maximum CDW, as a 50% reduction already decreased the final CDW. When available carbon is increased, the standard concentration of magnesium in CGXII is limiting, as we observed a further increase in CDW when supplying more magnesium under this condition. A previous study also showed that even under reduced carbon concentrations magnesium may limit biomass formation in *C. glutamicum* (Yang et al., 2021). We observed no effects by omitting PCA, as neither growth rate nor lag phase nor final CDW were affected, these results are in agreement with previous findings (Yang et al., 2021). However, in other studies increased lag-phases or decreased growth rates have been observed when PCA is omitted, indicating that other factors probably play a role as well (Unthan et al., 2014; Graf et al., 2019). Omission of biotin led to a reduced final CDW as has been observed previously (Peters-Wendisch et al., 2014) and is expected as *C. glutamicum* is biotin auxotrophic.

While the model overall had a non-significant lack-of-fit, a verification of the predicted optimum point did lead to lower astaxanthin titers overall. This is probably due to the model being heavily skewed because the low concentrations lead to reduced or no growth, thus also leading to strongly reduced astaxanthin titers. Except for the complete omission of compounds like magnesium, calcium and biotin there was no observable trend for these compounds. The only component showing a more variable behavior was the trace metal solution, where there appeared to be actual curvature in the data.

Therefore, the contained metal salts were tested separately to verify their singular effects. These results showed that iron is absolutely necessary for growth which is in accordance with literature (Nakayama et al., 1964). Zinc is also important but to a lesser degree, with maximum CDW already being reduced at 50% of the standard zinc concentration and further reduced but growth still being possible in the complete absence of zinc. Previous analyses have shown that zinc is the most abundant metal in *C. glutamicum* cells when grown in CGIII complex medium, indicating high requirements, high uptake rates in general, or both (Liebl, 2005). The fact that a reduction in zinc in the medium by 50% already leads to a reduced maximum CDW seems to indicate a high demand for zinc.

The other trace metals showed no limiting influence on the maximum CDW in the first cultivation, however upon transferring cells grown in manganese-free medium to a second round of manganese-free medium we observed a significant reduction in maximum CDW, indicating severe limitation and the essentiality of this metal. One of the known Mn-dependent enzymes of *C. glutamicum* is the superoxide dismutase, which has been shown to not accept Fe or other transition metals (El Shafey et al., 2008). For copper, no growth phenotype was observed as even in a second round of cultivation in Cu-free medium maximum CDW was unimpeded. However, astaxanthin titers were significantly reduced in the second cultivation showing that copper is necessary for processes other than growth (Supplementary Figure S5).

In regard to astaxanthin formation, we observed the majority of the effects from manganese, iron and their interaction. Both iron and manganese regulation have been studied in *C. glutamicum* in the form of the regulators DtxR and RipA (Wennerhold and Bott, 2006; Küberl et al., 2020; Müller et al., 2020), as well as MntR (Baumgart and Frunzke, 2015), respectively. The MntR regulon is overall a lot simpler than DtxR/RipA, as it primarily regulates expression of a putative manganese transporter gene called *mntT* (cg1623) and deletion of either *mntR* or *mntT* showed no distinct phenotype. A second regulator that is putatively under MntR control is a MarR-type regulator of unknown function (cg0343) and another potentially Mn-binding DtxR type regulator (cg2784) exists as well. However, these have not been characterized further. Among the differentially regulated genes observed upon deletion of *mntR* (Baumgart and Frunzke, 2015), the only one with a possible indirect link to carotenoid biosynthesis is the arginine biosynthetic operon. As arginine biosynthesis has high requirements for redox-equivalents, i.e. 4 mol/mol NADPH (Zhan et al., 2019) this might have an effect on the final steps of astaxanthin biosynthesis as both β-carotene hydroxylation and ketolation, as well as the entire carotenoid biosynthesis pathway, are dependent on 20 mol/mol of NAD(P)H to convert GAP and pyruvate to lycopene and another 6 mol/mol NAD(P)H to oxidize it to astaxanthin. In *Streptococcus sanguinis* manganese-starvation was also shown to have wide-ranging influence via the small alarmone molecule ppGpp (Puccio et al., 2020) and effects from this molecule on carotenoid biosynthesis in *C. glutamicum* have also been described (Ruwe et al., 2019). However, we only observed a positive effect on the biosynthesis of decaprenoxanthin and not β-carotene which makes it less likely that there is a general response in the synthesis of carotenoid precursors, and presumably also not via small alarmone molecules like ppGpp.

The effects of an increased iron supply were very positive for the synthesis of astaxanthin. Both terminal enzymes converting β-carotene to astaxanthin are iron-dependent (Fraser et al., 1997) which could present a reasonable explanation. Additionally, it is possible that these enzymes are capable of binding manganese as well, which could lead to a reduced or even complete cessation of function. This behavior has been observed in several hydroxylases, e.g., methane hydroxylase (Atta et al., 1993) or proline hydroxylase (Lawrence et al., 1996). In fact, Fraser et al. tested various potential cofactors for β-carotene hydroxylase and ketolase CrtZ and CrtW by adding NAD^+^ but also the metal ions Mn^2+^ or Mg^2+^ to their enzyme assays. They observed no positive and often even inhibitory effects on enzyme activity, however no details are given on which additives gave which effect (Fraser et al., 1997). Thus, it could be speculated that a possible mismetallation with Mn^2+^ instead of Fe^2+^ leads to a reduction in enzyme activity and thus a reduction in astaxanthin biosynthesis. This is well in agreement with the fact that we observed a synergistic effect between low manganese and high iron concentrations. This mismetallation could also affect other enzymes, e.g., aconitase, for which this has been observed in mitochondria (Zheng et al., 1998), and thus explain the same synergistic effects observed in this study for the maximum CDW. Furthermore, the effects from iron concentration on astaxanthin biosynthesis are well in agreement with our previous study on improving fermentation conditions for astaxanthin production. The most important factor we identified was an alkaline pH, as well as a low aeration rate. At acidic pH 6 a complete cessation of xanthophyll formation was observed (Meyer et al., 2023) and it has previously been shown that a link between acidic pH and iron starvation exists in *C. glutamicum* (Follmann et al., 2009)*.* Consequently, a better iron supply for the cells at basic pH would be expected. A recent study by Müller et al. also showed a link between dissolved CO_2_ and its positive influence on the reduction of Fe^3+^ to Fe^2+^ when it is bound to phenolic compounds like PCA (Müller et al., 2020). Taken together, it can be hypothesized that the observed results from the fermentation optimization are at least partially due to an improved availability of iron for the cells (Meyer et al., 2023). Furthermore, the effect of CO_2_ on iron reduction could also explain the positive effect that urea showed on astaxanthin formation, as its cleavage releases both two molecules of NH_3_ as well as one molecule of CO_2_. Contrary to this, PCA showed no effect on astaxanthin formation in our experiments, which is surprising, if it is involved in both iron uptake (Liebl et al., 1989) as well as iron reduction (Müller et al., 2020). However, its role and importance as an iron uptake facilitator is still being discussed (Unthan et al., 2014).

The optimization experiments also showed quite varied results regarding the intermediate carotenoids of astaxanthin biosynthesis. For a strain only expressing the ketolase gene *crtW*, the optimized trace salts primarily influence the conversion from echinenone to canthaxanthin. The titer of the ketolated carotenoids canthaxanthin and echinenone taken together only increases by 13.7% and 29.2% in CGXII and CGAST1, respectively, when using the optimized trace salts. However, the percentage of canthaxanthin among the ketolated products increases from 17.5% to 69.0% and 24.8%–69%. This also coincides with a decrease in total carotenoids by 62% and 55% in CGXII and CGAST1, respectively. A strain expressing just the hydroxylase gene *crtW* showed a somewhat different behavior. The concentration of the zeaxanthin precursor β-cryptoxanthin was overall far lower compared to echinenone, so most of the increase in zeaxanthin comes from β-carotene being hydroxylated twice. Still, while the concentration of hydroxylated carotenoids was higher than that of their ketolated equivalents in the other strain, the inhibitory effect on the total carotenoid production was far less pronounced. Similar results can be seen in the strain producing astaxanthin, where the titers of astaxanthin increases significantly, e.g., by 4.6x in CGAST1 medium, but the total carotenoids only decrease by 29% when compared to the initial trace metal composition.

That the formation of xanthophylls has some kind of feedback inhibition on β-carotene synthesis has been described often in literature, however the mechanism is yet unknown (Zhang et al., 2018; Basiony et al., 2022; Göttl et al., 2023). Accordingly, in this work, when increasing xanthophyll titers by media optimization the total amount of carotenoids produced was significantly reduced. However, there is a clear difference in the degree of inhibition that occurs, as ketolated xanthophylls clearly lower total carotenoid production more than either of their hydroxylated counterparts and even astaxanthin. Furthermore, the twice ketolated canthaxanthin shows a much stronger effect than the single ketolated intermediate echinenone.

A possible explanation could be that there is no direct feedback inhibition on enzymes in the carotenoid biosynthesis pathway, but that the changes stem from changes in membrane fluidity and potentially subsequent enzyme inhibition by this change. Gruszecki and Strzałka (2005) as well as Sujak (2009) reviewed the effect of various carotenoids on membrane fluidity. The latter especially reviewed the toxicity shown by the interaction of canthaxanthin with model membranes which could explain the severe effect its accumulation has on carotenoid formation.

Finally, it was possible to transfer the optimized trace metals into a 2 L fed-batch fermentation process. While the control process reached titers of astaxanthin comparable to our previously published process optimization (Meyer et al., 2023), the optimized trace metals could boost this titer 2.8-fold to 176 mg L^−1^. This is the highest astaxanthin titer achieved with *C. glutamicum* so far and exceeds our previous titer of 103 mg L^−1^, which was reached with a further metabolically engineered strain (Göttl et al., 2024). Productivity and yield were also increased from 1.5 mg L^−1^ h^−1^ to 2.44 mg L^−1^ h^−1^ and 0.34 mg g^−1^ glucose to 0.58 g g^−1^ glucose (Göttl et al., 2024). While the achieved production titers are still lower than those with e.g., *E. coli* (1.18 g L^−1^) (Gong et al., 2020) our results show that there is a large potential in using *C. glutamicum* as a production host for carotenoids and that significant improvements are possible through other means than metabolic engineering. It might also be possible to increase these titers by further optimizing the media composition for the fermentative scale; however, our transfer of the trace metal composition from the milliliter to the liter scale showed very comparable increases to the previous extensive DoE results. As such, and taking into mind the high amount of work and resources required to further optimize the fermentation process compared to performing the experiments in the smaller scale and then upscaling them, it is unclear if investing such an amount of additional work would lead to results that are proportional to this investment. Furthermore, comparing these increases to other astaxanthin production processes where DoE methods have been applied, our increases are generally higher than described e.g., for *P. rhodozyma* (92% increase) (Ramírez et al., 2001) or *Chlorella zofingiensis* (74% increase) (Chen and Wang, 2013). Finally, these results can potentially be applied for other astaxanthin production processes as well.

## Data Availability

The original contributions presented in the study are included in the article/Supplementary Material, further inquiries can be directed to the corresponding author.
