# Supplementary material for: Response surface-based media optimization for astaxanthin production in Corynebacterium glutamicum
Source: Front Bioeng Biotechnol. 2025 Mar 11;13:1516522. doi: 10.3389/fbioe.2025.1516522 (PMC11933003; doi:10.3389/fbioe.2025.1516522)
Supplement: Supplementary file 2 [file DataSheet1.docx]

Supplementary Material

Supplementary Table S1. Overview over the effects of main media components on cell dry weight and astaxanthin titers. The effect of each factor on the response, the corresponding F-value and the result of the ANOVA analysis are given as well. Factors separated by a colon indicate two-factor interactions while squared factors indicate quadratic effects.

|  | CDW | | Astaxanthin | |
| --- | --- | --- | --- | --- |
| Factor | *t*-value | Prob > *t* | *t*-value | Prob > *t* |
| Intercept | 7.01 | <0.001 | 13.85 | <0.001 |
| Block | -4.80 | <0.001 | -8.11 | <0.001 |
| Acetate | 6.84 | <0.001 | 14.50 | <0.001 |
| Glucose | 5.02 | <0.001 | 10.59 | <0.001 |
| Ammonium sulfate | 0.87 | 0.39 | -1.28 | 0.21 |
| Urea | 2.57 | 0.01 | 14.30 | <0.001 |
| Phosphate | -0.72 | 0.47 | 5.20 | <0.001 |
| Acetate:Glucose | 3.37 | <0.01 | 6.13 | <0.001 |
| Acetate:Ammonium sulfate | 1.56 | 0.12 | 0.55 | 0.59 |
| Acetate:Urea | -2.03 | 0.05 | -2.19 | 0.03 |
| Acetate:Phosphate | -0.79 | 0.43 | 2.33 | 0.02 |
| Glucose:Ammonium sulfate | 3.23 | <0.01 | 0.10 | 0.92 |
| Glucose:Urea | 3.49 | <0.001 | 7.45 | <0.001 |
| Glucose:Phosphate | -2.17 | 0.03 | 0.10 | 0.92 |
| Ammonium sulfate:Urea | 0.59 | 0.56 | 1.44 | 0.15 |
| Ammonium sulfate:Phosphate | -1.89 | 0.06 | -0.03 | 0.97 |
| Urea:Phosphate | 1.51 | 0.14 | -0.94 | 0.35 |
| Acetate^2^ | -0.60 | 0.55 | -6.24 | <0.001 |
| Glucose^2^ | -0.58 | 0.56 | -9.19 | <0.001 |
| Ammonium sulfate^2^ | -0.26 | 0.80 | 0.31 | 0.76 |
| Urea^2^ | -0.47 | 0.64 | -1.53 | 0.13 |
| Phosphate^2^ | 0.07 | 0.94 | 0.64 | 0.53 |
|  | F-value | Prob > F | F-value | Prob > F |
| Lack of fit | 2.36 | 0.04 | 3.07 | 0.01 |
| First order | 15.07 | <0.001 | 118.37 | <0.001 |
| Second order | 5.21 | <0.001 | 9.40 | <0.001 |
| Quadratic terms | 0.35 | 0.88 | 36.02 | <0.001 |

Supplementary Table S2. RSM for the effects of various trace metals on astaxanthin production and CDW in *C. glutamicum* ASTA*. The effect of each factor on the response, the corresponding F-value and the result of the ANOVA analysis are given as well. Factors separated by a colon indicate two-factor interactions while squared factors indicate quadratic effects.

|  | CDW | | Astaxanthin | |
| --- | --- | --- | --- | --- |
| Factor | F-value | Prob > F | F-value | Prob > F |
| Intercept | 7.01 | <0.001 | 38.02 | <0.001 |
| FeSO_4_ | 6.84 | <0.001 | 14.32 | <0.001 |
| MnSO_4_ | 5.02 | <0.001 | -6.54 | <0.001 |
| ZnSO_4_ | 0.87 | <0.001 | 5.13 | <0.001 |
| CuSO_4_ | 2.57 | <0.001 | -1.00 | 0.33 |
| NiCl_2_ | -0.72 | <0.001 | -2.06 | 0.05 |
| FeSO_4_ : MnSO_4_ | 3.37 | 0.00 | -2.32 | 0.03 |
| FeSO_4_ : ZnSO_4_ | 1.56 | 0.12 | -0.14 | 0.89 |
| FeSO_4_ : CuSO_4_ | -2.03 | 0.05 | 0.62 | 0.55 |
| FeSO_4_ : NiCl_2_ | -0.79 | 0.43 | 1.14 | 0.27 |
| MnSO_4_ : ZnSO_4_ | 3.23 | 0.00 | 0.10 | 0.92 |
| MnSO_4_ : CuSO_4_ | 3.49 | <0.001 | 1.49 | 0.15 |
| MnSO_4_ : NiCl_2_ | -2.17 | 0.03 | 0.34 | 0.74 |
| ZnSO_4_ : CuSO_4_ | 0.59 | 0.56 | -0.26 | 0.80 |
| ZnSO_4_ : NiCl_2_ | -1.89 | 0.06 | -0.51 | 0.61 |
| CuSO_4_ : NiCl_2_ | 1.51 | 0.14 | 1.02 | 0.32 |
| FeSO_4_^2^ | -0.60 | 0.55 | -7.01 | <0.001 |
| MnSO_4_^2^ | -0.58 | 0.56 | -0.38 | 0.71 |
| ZnSO_4_^2^ | -0.26 | 0.80 | -8.19 | <0.001 |
| CuSO_4_^2^ | -0.47 | 0.64 | -0.86 | 0.40 |
| NiCl_2_^2^ | 0.07 | 0.94 | -2.08 | 0.05 |
|  | F-value | Prob > F | F-value | Prob > F |
| Lack of fit | 2.36 | 0.04 | 5.16 | 0.01 |
| First order | 15.07 | <0.001 | 55.88 | <0.001 |
| Second order | 5.21 | <0.001 | 1.08 | 0.43 |
| Quadratic terms | 0.35 | 0.88 | 26.08 | <0.001 |


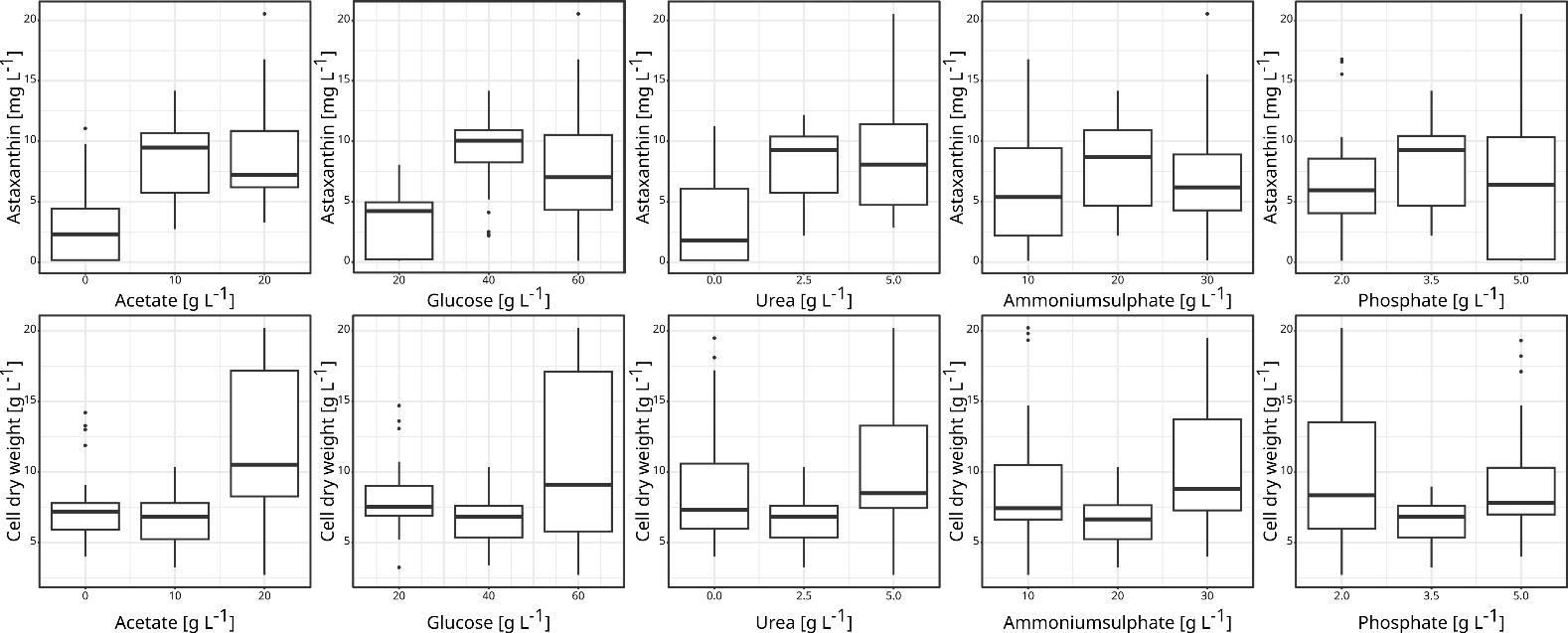


Supplementary Figure S1. Boxplot of the macronutrient optimization experiment with *C  glutamicum* ASTA* in CGXII medium at 30°C, 1100 rpm in a BioLector FlowerPlate. Concentrations are given as mg L^-1^ or g L^-1^. Outliers are shown as dots.


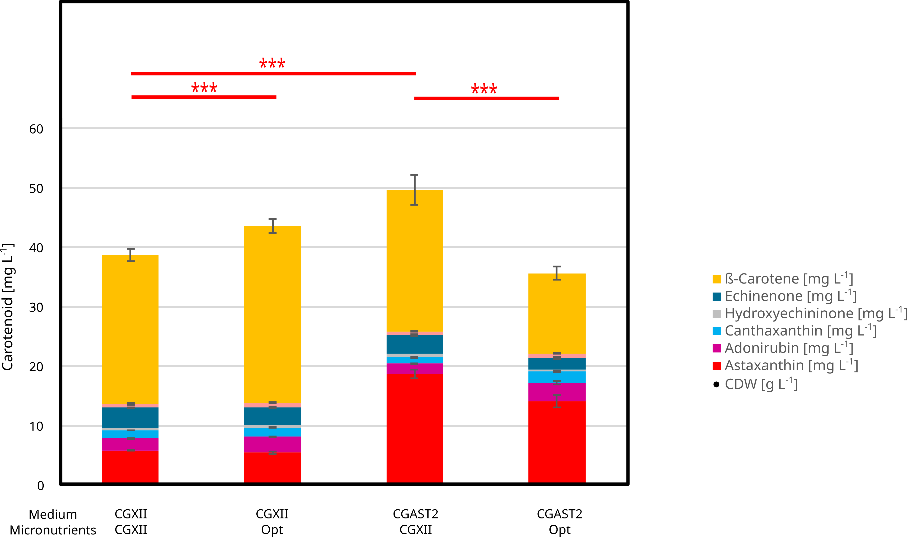


Supplementary Figure S2. Verification experiment in 10 mL flasks using strain *C. glutamicum* ASTA*. The base medium used was either unmodified CGXII or CGAST2.The micronutrient composition was optimized according to the results of the DoE and contained either the normal CGXII composition or 360 mg L^-1^ MgSO_4_ x 7 H_2_O (1.44 fold the CGXII concentration), 12.7 mg L^-1^ CaCl_2_ (1.27 fold) and 1.49 fold the initial amount of the trace metal solution. The cultivation was performed at 120 rpm and 30°C in the dark for 48 h. The standard deviations are shown as error bars and significances are given for the astaxanthin titers.


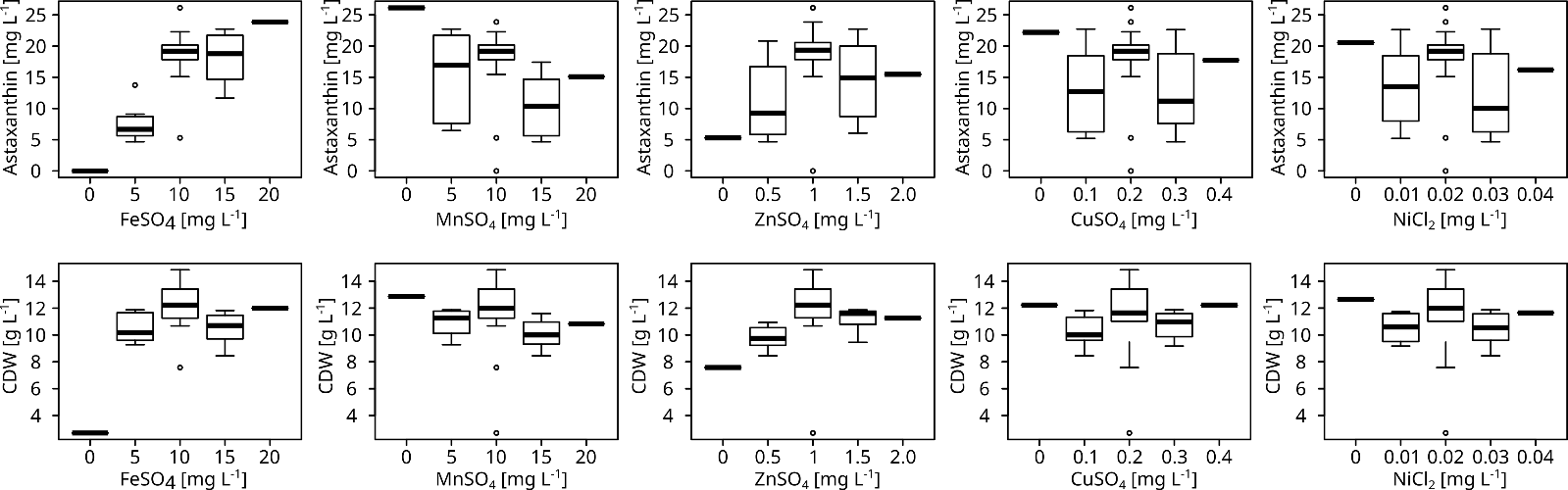


Supplementary Figure S3. Boxplot of the trace metal optimization experiment with *C. glutamicum* ASTA* in modified CGXII medium at 30°C, 1100 rpm in a BioLector FlowerPlate. Concentrations are given as mg L^-1^ or g L^-1^. Outliers are shown as dots.


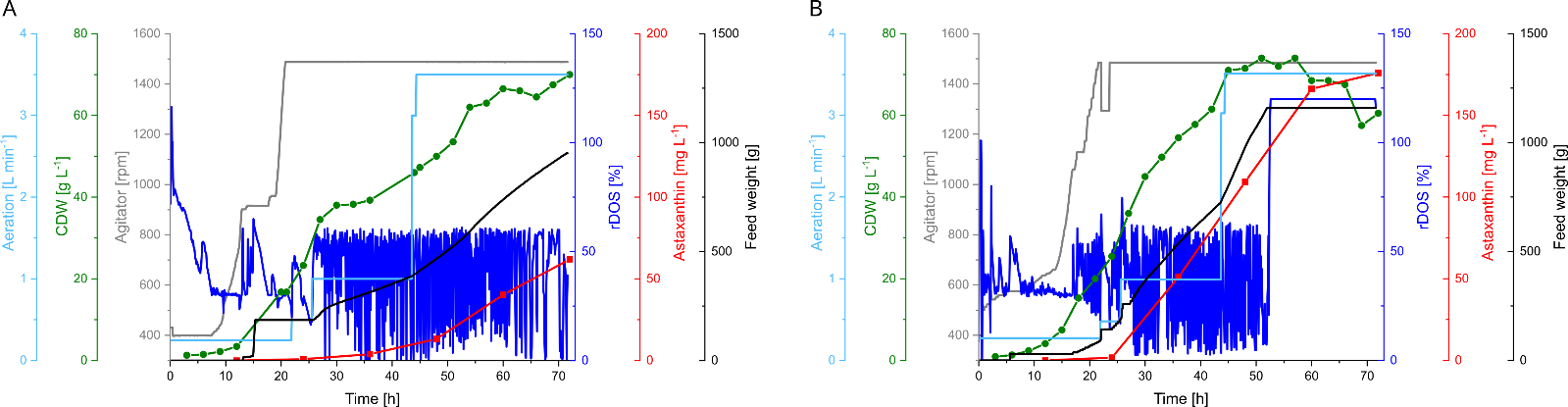


Supplementary Figure S4. Fermentations of strain *C. glutamicum* ASTA* in a 2 L fed-batch reactor using 1 L of a modified Knoll HCDC medium with either the normal trace metal composition (A) or the composition optimized for microcultivation (B). A feed of 1 L 600 g L^-1^ glucose was added based on the pO2 signal. The process was performed at 30°C and pH 8 using 25% (v v^-1^) ammonia and 10% (v v^-1^) phosphoric acid.


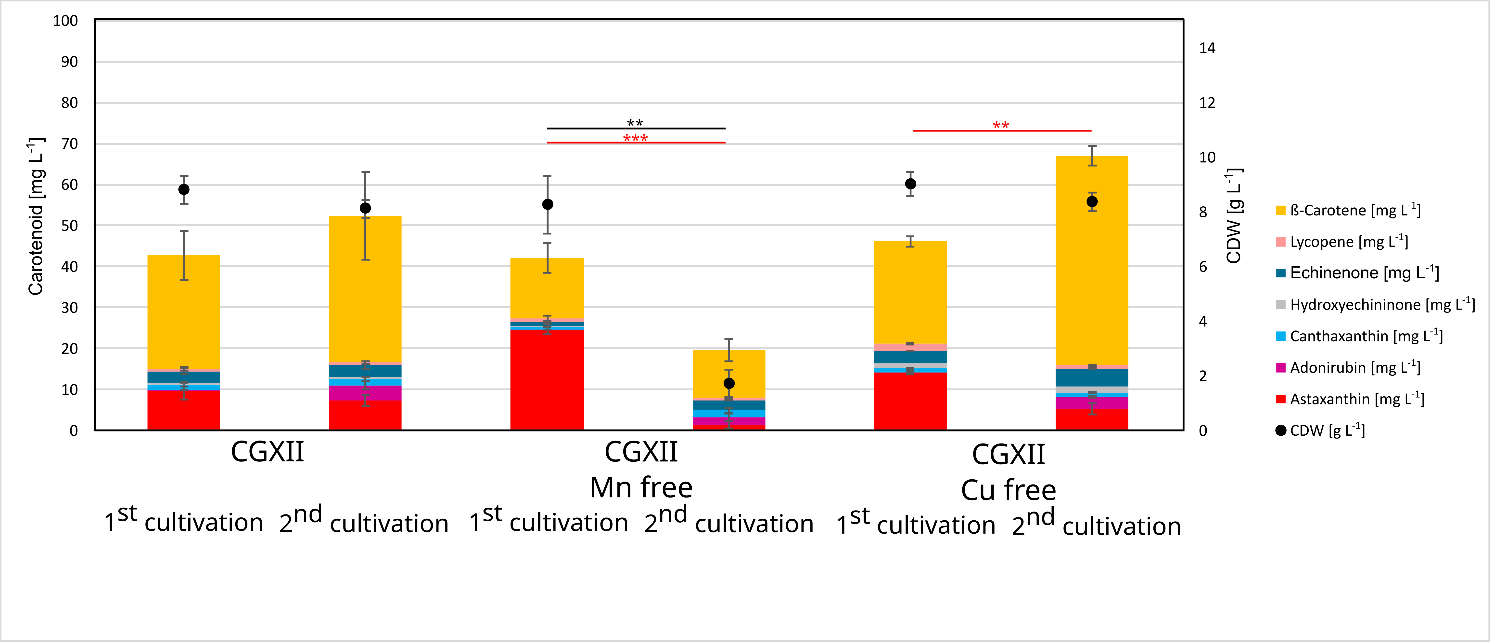


Supplementary Figure S5. Repeated cultivation experiment in 10 mL flasks with strain *C. glutamicum* ASTA* in CGXII medium with either the default composition or in medium without manganese or copper. The second cultivation flasks were inoculated from the first cultivation of the same medium after 24 h to OD 1. All flasks were harvested at the same point after 96 h or 120 h, respectively. The experiments were performed in triplicates and performed at 30 °C and 120 rpm in the dark. Standard deviations and significances calculated via Student’s t-test are shown, the latter only for biomass and astaxanthin titers.
